# Selenite reduction by the obligate aerobic bacterium *Comamonas testosteroni* S44 isolated from a metal-contaminated soil

**DOI:** 10.1186/s12866-014-0204-8

**Published:** 2014-08-07

**Authors:** Shixue Zheng, Jing Su, Liang Wang, Rong Yao, Dan Wang, Yujia Deng, Rui Wang, Gejiao Wang, Christopher Rensing

**Affiliations:** 1 State Key Laboratory of Agricultural Microbiology, College of Life Science and Technology, Huazhong Agricultural University, Wuhan 430070, PR China; 2 Department of Plant and Environmental Sciences, Faculty of Sciences, University of Copenhagen, Thorvaldsensvej 40, Frederiksberg C, 1871, Denmark; 3 Department of Chemistry, University of Copenhagen, Universitetsparken 5, Copenhagen Ø, 2100, Denmark; 4 Tobacco Company of Enshi, Hubei Province, Enshi 445000, Hubei, PR China

**Keywords:** Se(IV) reduction, Selenium nanoparticles (SeNPs), Resistance to heavy metals and metalloids, iscR, Se(VI) reduction

## Abstract

**Background:**

Selenium (Se) is an essential trace element in most organisms but has to be carefully handled since there is a thin line between beneficial and toxic concentrations. Many bacteria have the ability to reduce selenite (Se(IV)) and (or) selenate (Se(VI)) to red elemental selenium that is less toxic.

**Results:**

A strictly aerobic bacterium, *Comamonas testosteroni* S44, previously isolated from metal(loid)-contaminated soil in southern China, reduced Se(IV) to red selenium nanoparticles (SeNPs) with sizes ranging from 100 to 200 nm. Both energy dispersive X-ray Spectroscopy (EDX or EDS) and EDS Elemental Mapping showed no element Se and SeNPs were produced inside cells whereas Se(IV) was reduced to red-colored selenium in the cytoplasmic fraction in presence of NADPH. Tungstate inhibited Se(VI) but not Se(IV) reduction, indicating the Se(IV)-reducing determinant does not contain molybdenum as co-factor. Strain S44 was resistant to multiple heavy and transition metal(loid)s such as Se(IV), As(III), Cu(II), and Cd(II) with minimal inhibitory concentrations (MIC) of 100 mM, 20 mM, 4 mM, and 0.5 mM, respectively. Disruption of *iscR* encoding a transcriptional regulator negatively impacted cellular growth and subsequent resistance to multiple heavy metal(loid)s.

**Conclusions:**

*C. testosteroni* S44 could be very useful for bioremediation in heavy metal(loid) polluted soils due to the ability to both reduce toxic Se(VI) and Se(IV) to non-toxic Se (0) under aerobic conditions and to tolerate multiple heavy and transition metals. IscR appears to be an activator to regulate genes involved in resistance to heavy or transition metal(loid)s but not for genes responsible for Se(IV) reduction.

## Background

The essential trace elemental selenium (Se) is the 34th element on the periodic table and plays a fundamental role in human health [1]. Se is involved in several major metabolic pathways, such as thyroid hormone metabolism, antioxidant defense systems and immune function [2]. In humans, selenium has navigated a narrow range from dietary deficiency (<40 μg per day) to toxic levels (>400 μg per day) [3]. Selenium toxicity in humans has been reported in the Chinese provinces Hubei and Shaanxi and in Indian Punjab, where Se levels in locally produced foods were found to be very high (750–4990 μg per person and day) [4]. The variation of Se status in humans both related to either Se excess or deficiency largely depends on the diet consisting of various crops, vegetables, fruits and meat [1]. Therefore, it is essential to understand the factors controlling the dynamic distribution of Se in the environment. Microorganisms are involved in the transformation of selenium from one oxidation state to another [5]-[7]. A few studies reported that bacteria oxidized selenium to Se(IV) and Se(VI) in soils [8],[9]. The formation of volatile methylated selenium species was also studied in several bacteria [5],[7],[10]. In addition, numerous bacteria were shown to reduce Se(VI)/Se(IV) to elemental Se, visible as red-colored nano-selenium [11]-[16].

Se(IV)-reducing bacteria generate red-colored elemental selenium nanoparticles (SeNPs) either under aerobic or under anaerobic conditions. Anaerobic Se(IV)-reducing bacteria encompass *Thauera selenatis*[17], *Aeromonas salmonicida*[18] and purple non-sulfur bacteria [14]. Aerobic bacteria involved in Se(IV) reduction include diverse species such as *Rhizobium* sp. B1 [19], *Stenotrophomonas maltophilia* SeITE02 [11], *Pseudomonas* sp. CA5 [13], *Duganella* sp. and *Agrobacterium* sp. [20]. However, the exact mechanism of selenium metabolism and reduction is still far from being elucidated.

Some studies implied that diverse enzymes are involved in dissimilatory reduction based on the appearance of extracellular and/or intracellular SeNPs in different microbes [12],[21]. Three different pathways were suggested as to the molecular mechanisms underlying Se(IV) reduction so far. The periplasmic nitrite reductase was responsible for Se(IV) reduction in *T. selenatis*[17] and *Rhizobium selenitireducens*[22]. Another mechanism linking redox precipitation of both elemental sulfur and elemental selenium was observed outside sulfate-reducing bacterial cells. *Desulfomicrobium norvegicum* reduced sulfate to sulfide (S^2−^) through the sulfate reduction pathway and then released sulfide into the extracellular medium [23]. Glutathione (GSH) also reacts with Se(IV) to produce GS-Se-SG which will generate GS-Se^−^. This reaction is catalyzed by a GSH reductase in purple non-sulfur bacteria such as *Rhodospirillum rubrum* and *Rhodobacter capsulatus* under anoxic conditions [14],[24]. A GSH reductase was also potentially involved in Se(IV) reduction in *Pseudomonas seleniipraecipitans*[25]. Unfortunately, so far no gene product or enzyme solely responsible for Se(IV) reduction has been identified *in vivo*. Several enzymes were shown to be involved in Se(IV) reduction in different microbes, Se(IV) reduction took place either in the cytoplasm [11],[20],[21] or in the periplasm [17].

We had previously isolated an antimony-oxidizing bacterium, the strictly aerobe *Comamonas testosteroni* S44, from an antimony mine in Lengshuijiang, Hunan province, southern China [26]. A large number of genes encoding putative metal(loid) resistance proteins, mobile genetic elements (MGEs) and evidence of recent horizontal gene transfer (HGT) events indicate progressive adaption to this extreme environment [26].

In this study, we investigated the process of Se(IV) reduction leading to biosynthesized nanoparticles under aerobic condition by Scanning Electron Microscopy (SEM), Transmission Electron Microscopy (TEM) and Electron Dispersion Spectroscopy (EDS) Elemental Mapping. In addition, transposon mutagenesis was employed to identify genes responsible for selenium resistance and reduction.

## Results

### *C. testosteroni* S44 was able to reduce Se(IV) under aerobic condition

**Figure 1:**
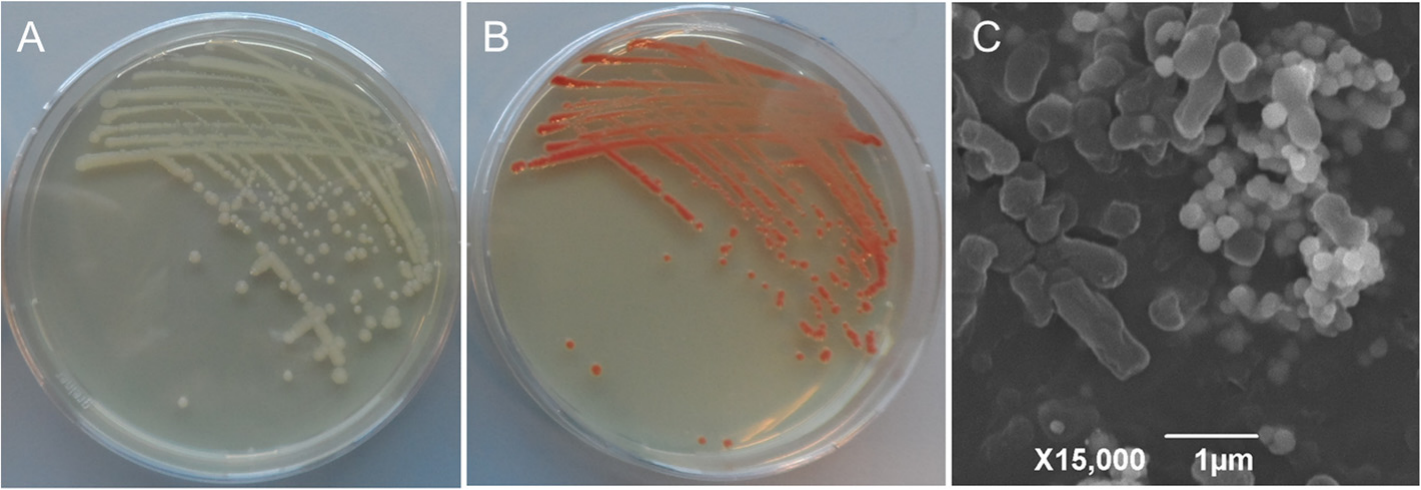
***C. testosteroni*S44 reduced selenite to red elemental SeNPs.** Growth of *C. testosteroni* S44 on LB plates without **(A)** or with 1.0 mM sodium selenite **(B). (C)** SEM image of *C. testosteroni* S44 cells amended with 20 mM sodium selenite, showing round elemental SeNPs and rod-shaped bacterial cells.

Initial growth experiments confirmed that *C. testosteroni* S44 was not able to grow under anaerobic condition indicating it is an obligate aerobe. In addition, *C. testosteroni* S44 reduced Se(IV) to elemental selenium that formed red nanoparticles under aerobic condition (Figure 1). These red-colored SeNPs were very stable in the supernatant or on solid plates at room temperature. They were still visible after sterilization at 121°C for 30 min.

**Figure 2:**
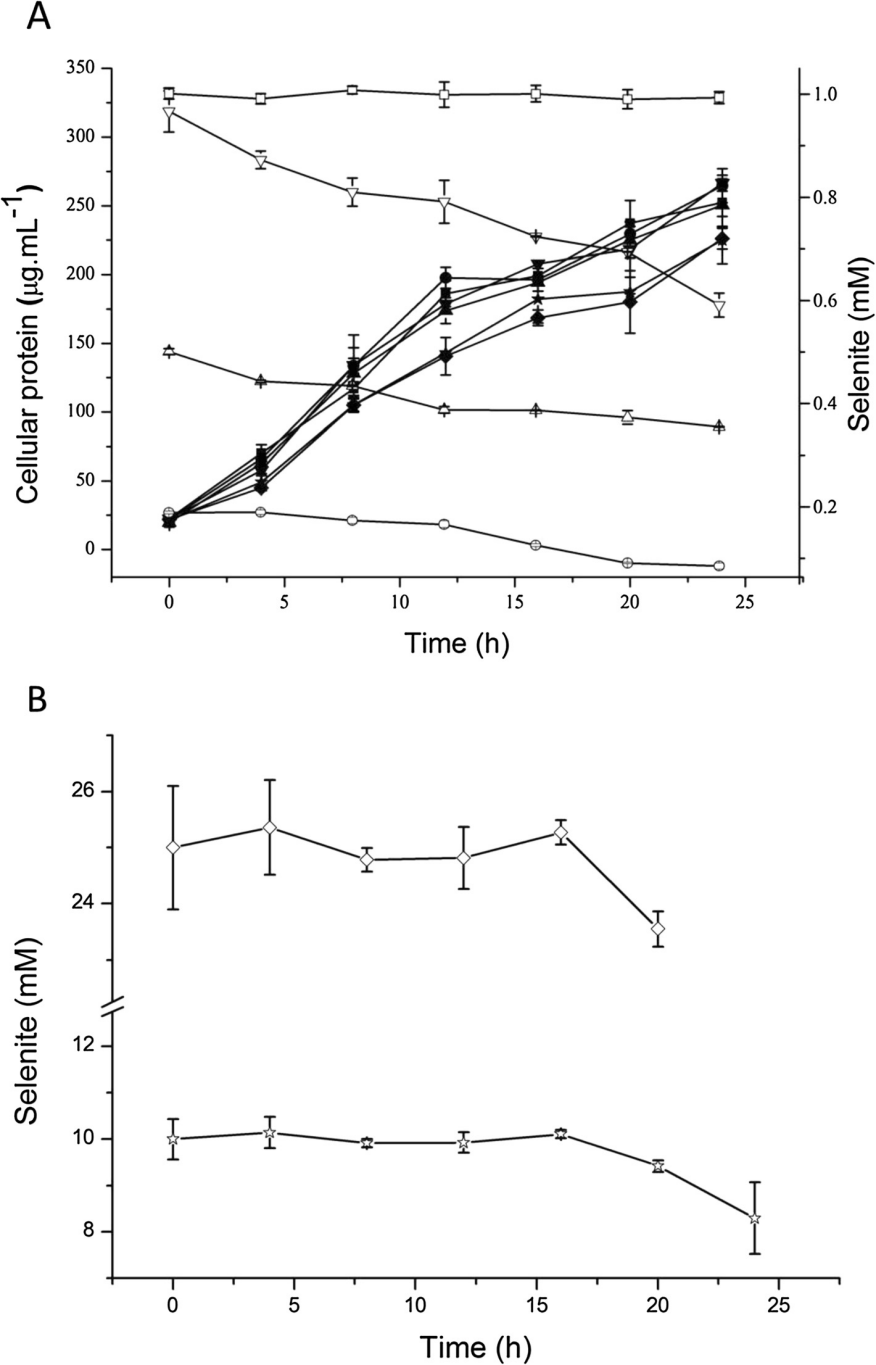
**Growth and Se(IV)-reduction of*C. testosteroni*S44 in LB broth with different concentrations of sodium selenite.** Filled symbols show strain *C. testosteroni* S44 grown at 0.0 mM (■), 0.2 mM (●), 0.5 mM (▲), 1.0 mM(▼), 10.0 mM(★), and 25.0 mM (◆) sodium selenite **(A)**. Open symbols show sodium selenite reduction at 1.0 mM (□) (control, no bacteria), 0.2 mM (○), 0.5 mM(△) and 1.0 mM (▽) sodium selenite **(A)**, as well as 10.0 mM (☆) and 25.0 mM (◇) sodium selenite **(B)**.

MICs for Se(IV) ranged from 100 mM to 150 mM in LB. Incubation in LB broth with less than 1.0 mM Se(IV) did not significantly affect growth of *C. testosteroni* S44 whereas it did negatively affect growth at concentrations above 10.0 mM Se(IV) (Figure 2). The broth obtained a weak orange color after 10 h incubation. Se(IV) was reduced by a biological rather than chemical process because no Se(IV) reduction was observed in the broth without the addition of bacterial cells. Strain S44 was unable to reduce the entire Se(IV) to elemental selenium both at low and at high Se(IV) concentrations. *C. testosteroni* S44 was only able to reduce 0.2 mM Se(IV) to 0.1 mM, 0.5 mM to 0.35 mM, 1.0 mM to 0.6 mM, 10.0 mM to 7.5 mM, and 25.0 mM to 20.7 mM remaining Se(IV), respectively during 24 h incubation in LB broth under aerobic condition (Figure 2).

### Characterization of SeNPs produced by *C. testosteroni* S44

**Figure 3:**
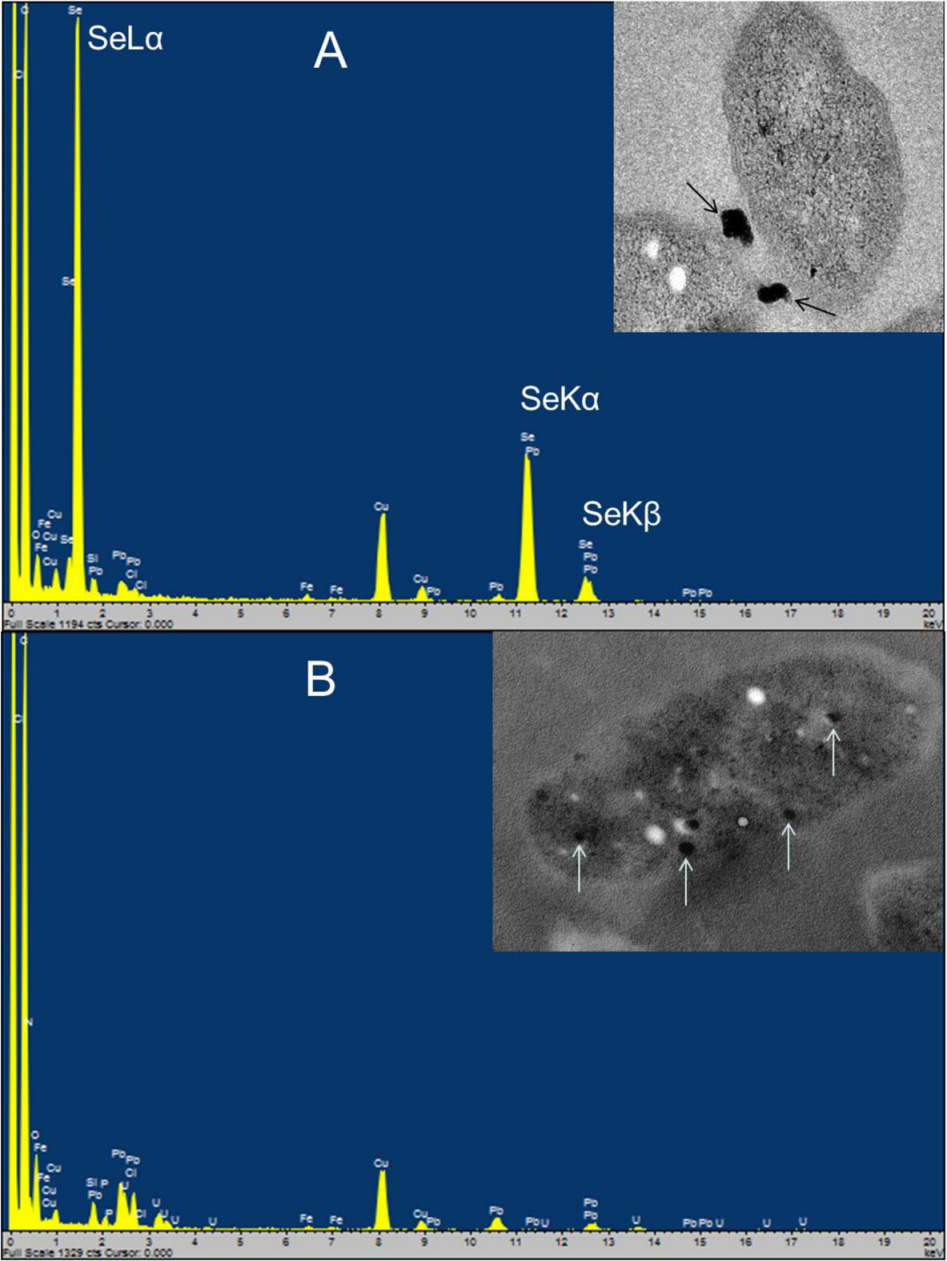
**EDX analysis of electron dense particles formed by cultures of*C. testosteroni*S44 amended with 1.0 mM sodium selenite. (A)** Extracellular particles pointed out by arrows. The emission lines for selenium are shown at 1.37 keV (peak SeLα), 11.22 keV (peak SeKα) and 12.49 keV (peak SeKβ). **(B)** Intracellular particles pointed out by arrows. No emission peaks of Se.

**Figure 4:**
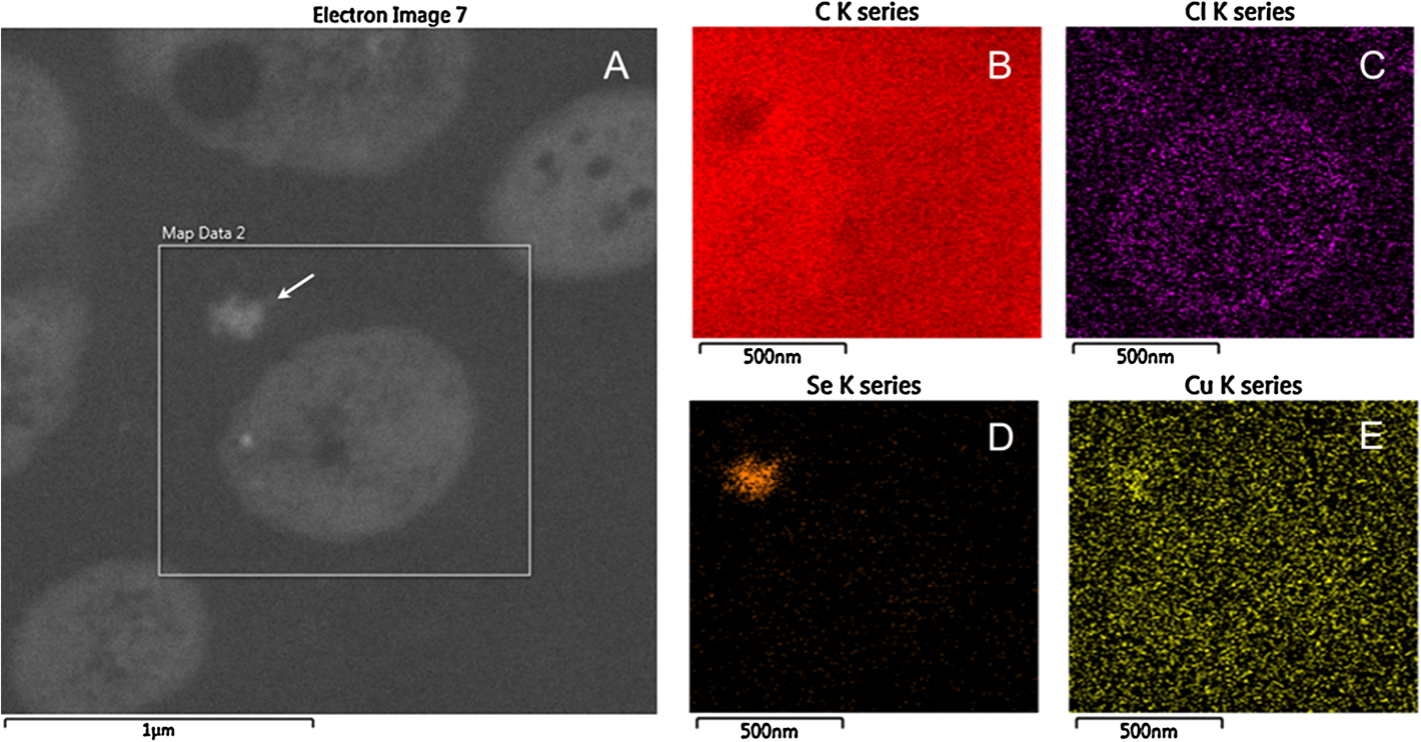
**Localization of selenium particles using EDS Elemental Mapping. (A)** The box showed the mapping area of B-E, where the K series peaks of the elements was used for mapping. The arrow points to an extracellular selenium particle. **B**, **C**, **D** and **E** show the distribution of different elements of C (from cell and Epon), Cl, Se and Cu (from Cu grids), respectively.

*C. testosteroni* S44 reduced Se(IV) to red colored SeNPs when grown in different media such as LB, TSB or CDM medium, with concentrations ranging from 0.20 to 50 mM Na_2_SeO_3_. The size of nanoparticles outside of cells ranged from 100 nm to 200 nm as judged from analysis of SEM photos (Figure 1C). The observed nanoparticles consisted of elemental selenium as determined by TEM- energy dispersive X-ray spectroscopy (EDX or EDS) analysis because the EDX spectrum of electron dense particles showed the expected emission peaks for selenium at 1.37, 11.22, and 12.49 keV corresponding to the SeLα, SeKα, and SeKβ transitions, respectively (Figure 3A). This strongly indicated Se(IV) was first reduced to elemental selenium. There was no obvious difference in intracellular morphology between *C. testosteroni* S44 amended with Se(IV) and the control without added Se(IV) during log phase or stationary phase (Additional file 1: Figure S1). We also did not observe emission peaks of elemental selenium from the spectrum of TEM-EDX based on suspected Se-particles in cells (Figure 3B). This indicated there were no selenium particles inside of the cells. To further investigate the distribution of selenium inside and outside of *C. testosteroni* S44 cells, EDS Elemental Mapping was used to detect selenium localization producing elemental maps showing the composition and spatial distribution of different elements in an unknown sample. Four elemental maps of carbon, chlorine, selenium and copper were obtained and shown in different colors based on the scanning area encompassing both the inside and outside of *C. testosteroni* S44 cells (Figure 4). The color of background was black in all elemental maps. The map of elemental chlorine (Cl) clearly showed the cell shape, distribution and density of Cl in the cell (Figure 4C). In contrast, elements carbon (C) (Figure 4B) and copper (Cu) (Figure 4E) were distributed both inside and outside of cells because cells were embedded by carbon-contained plastic Epon before section in order to maintain the cell shape, as well as sectional samples were coated by copper grids to support thin slicing of bio-samples. However, strong signals of selenium as shown by orange color were only observed outside of cells whereas the color in cells was black background even the white dots in cells suspected to be SeNPs were not similar to SeNPs outside of cells (Figure 4D), indicating that SeNPs were only formed outside of cells rather than inside of cells. The EDS map of elemental selenium was consistent with TEM-EDX result focusing on high density particles, i.e., SeNPs did not occur in the interior of *C. testosteroni* S44 cells. In addition, it was clear that small SeNPs aggregated into bigger particles outside of cells (Additional file 1: Figure S1).

### Tungstate inhibited Se(VI) but not Se(IV) reduction

**Figure 5:**
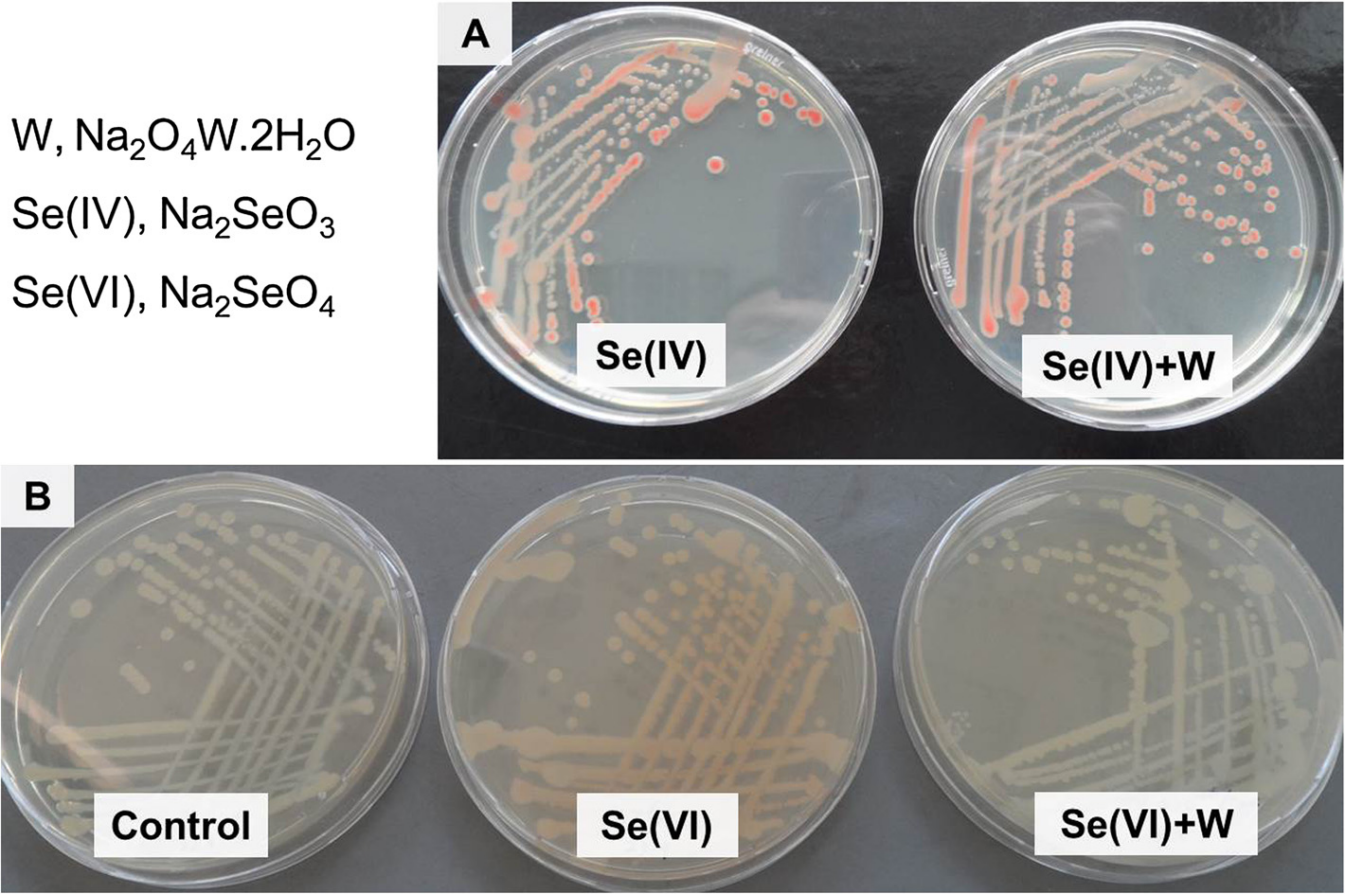
**Comparison of Se(IV) and Se(VI) reduction and tungstate inhibition in*C. testosteroni*S44.** Cultures were amended with 0.2 mM Se(IV) **(A)**, 5.0 mM Se(VI) **(B)**, respectively, and with or without 10 mM tungstate. Tungstate did not inhibit Se(IV) reduction both in CDM, LB and TSB media whereas it inhibited Se(VI) reduction in all media.

Tungsten has been used as an inhibitor of the molybdoenzymes, since it replaces molybdenum (Mo) in the Mo-cofactor (MoCo) of these enzymes. Tungstate did not affect reduction of Se(IV) (Figure 5A) since the same red color of the SeNPs could be observed whether tungstate was added to cells of *C. testosteroni* S44 or not. In contrast, addition of tungstate and Se(VI) resulted in no development of red colored nanoparticles as in the negative control with no added Se(VI) and tungstate. In contrast, addition of Se(VI) without tungstate resulted in red-colored colonies on LB agar plates (Figure 5B). Therefore, tungstate only inhibited molybdenum-dependent Se(VI) reduction and subsequent reduction to elemental selenium and formation of nanoparticles. Similar results were obtained in different media such as LB, TSB and CDM.

### The cytoplasmic fraction strongly reduced Se(IV) to SeNPs

**Figure 6:**
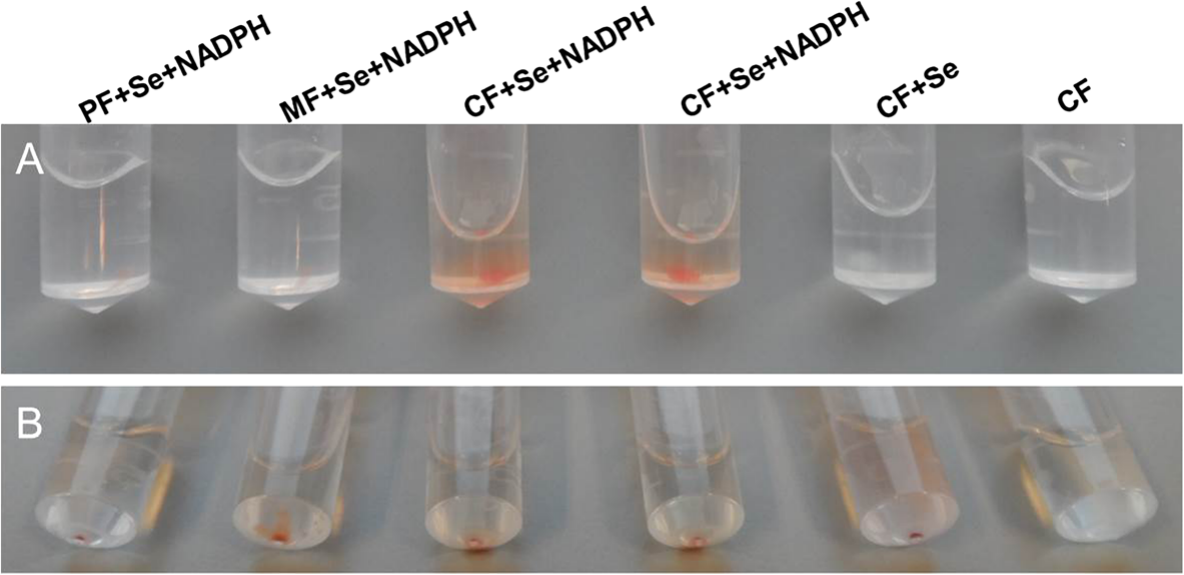
**Se(IV) reduction of cellular fractions amended with 0.2 mM Se(IV) and 0.2 mM NADPH at 24 h (A) and 5 days (B).** PF, periplasmic fraction; MF, membrane fraction; CF, cytoplasmic fraction.

To help determine how Se(IV) is reduced, different cellular fractions were isolated and the activity of Se(IV)-reduction was determined. Subcellular fractions were isolated after 12 h and 20 h growth in LB broth without Se(IV). 0.2 mM Se(IV) and 0.2 mM NADPH were added to different fractions at room temperature. After 24 h incubation, Se(IV) was reduced to red-colored selenium by the cytoplasmic fraction in the presence of NADPH whereas no red-colored selenium occurred in the cytoplasmic fraction without NADPH, indicating Se(IV) reduction was NADPH-dependent (Figure 6A). NADH gave the same results as NADPH. In contrast, periplasmic and membrane fractions were only able to reduce Se(IV) weakly. Even after an incubation for 5 days only a few red-colored SeNPs were observed (Figure 6B). Addition of Se(IV) to the cytoplasmic fraction (CF) but without NADPH also resulted in faint reddish-colored SeNPs after 5-days incubation, perhaps due to low amounts of residual NADPH left in the CF. In addition, fractions isolated from cells grown in medium with added Se(IV) had the same properties as fractions isolated from cells grown without Se(IV) in the medium suggesting that Se(IV) reduction was not induced by Se(IV).

### IscR is necessary for resistance of Se(IV) and other heavy or transition metal(loid)s but not for Se(IV) reduction

Approximately 10,000 transposon mutants were isolated and tested for Se(IV) resistance and reduction. Among these, 23 mutants showed lower resistance to Se(IV) and delayed Se(IV) reduction compared to the wild type. However, we did not find any mutant that did not reduce Se(IV) to red-colored selenium. The genomic regions flanking the transposon insertion of these 23 sensitive mutants were sequenced and analyzed by BlastX in the GenBank database. We selected four representative mutants as Tn5 was inserted into different positions of *iscR* in the two mutants of iscR-327 and iscR-513. Additionally, two other *iscR* Tn5-insertion mutants (iscR-280) and (iscS + 30) were obtained in another research project on microbial Sb(III) resistance and oxidation in our lab. The mutant iscR-327 displayed even lower resistance to Se(IV) than iscR-280 and iscR-513. *IscR* encodes a regulator of genes involved in iron-sulfur cluster genesis. Thus, these four mutants iscR-280, iscR-327, iscR-513 and iscS + 30 were selected for further study.

**Figure 7:**
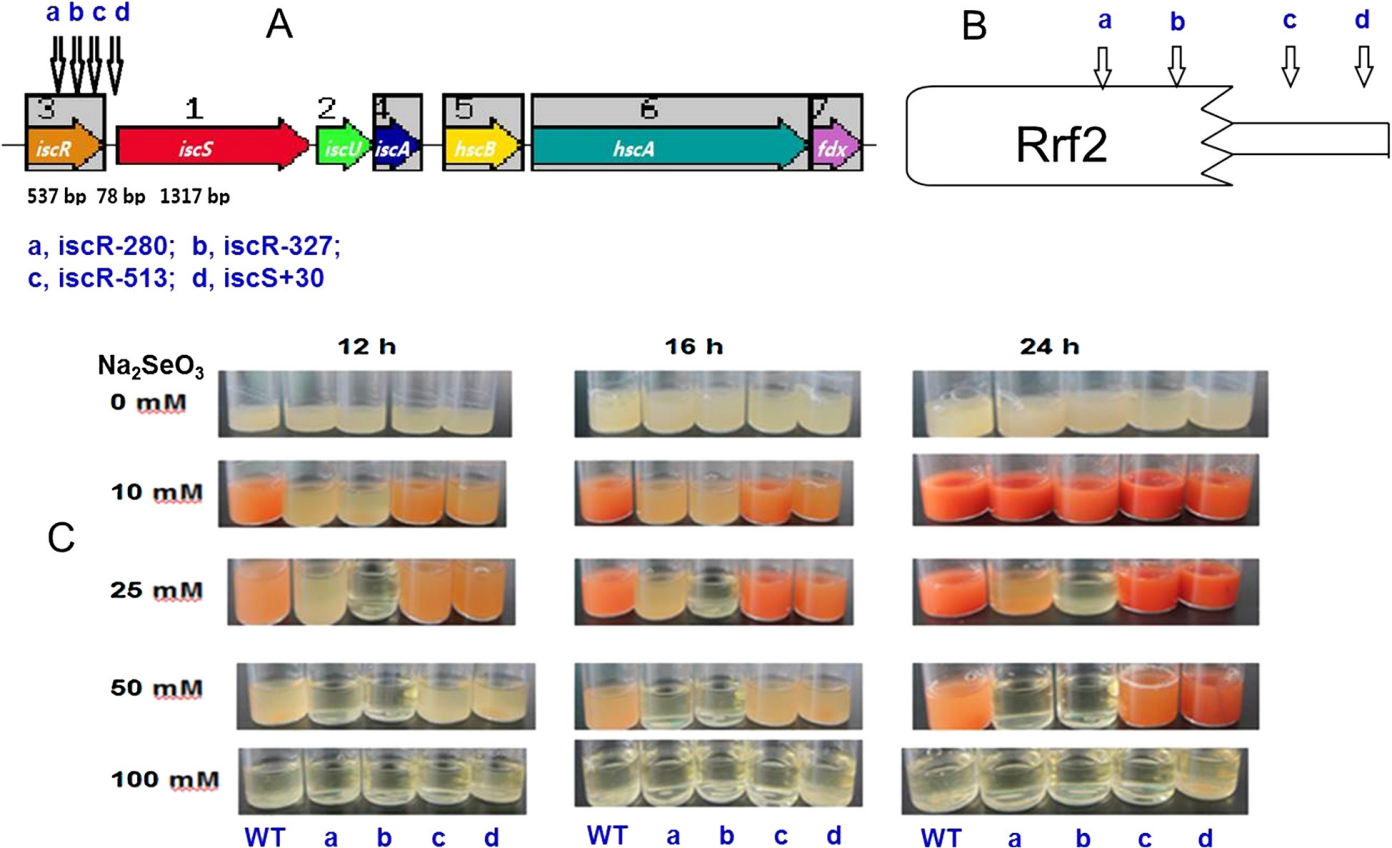
**Se(IV) resistance and reduction using different Se(IV) concentrations using four*iscR*insertional mutants in*C. testosteroni*S44.** The different sites of transposon insertions in *iscR* is given in nt from the translational start codon; +30 is an insertion upstream of *iscS***(A)**; the predicted domains of the IscR protein **(B)** and growth in LB medium amended with different concentrations of Se(IV) at different time points **(C)**. The four arrows indicate the four mutants of iscR-280 (a), iscR-327 (b), iscR-513 (c) and iscS + 30 (d), respectively in **A** and **B**. The order of the 5 PA bottles of **C** is wild type (WT), iscR-280 (a), iscR-327 (b), iscR-513 (c) and iscS + 30 (d), respectively.

The *isc* gene cluster contains *iscSUA-hscBA-fdx* in *C. testosteroni* S44 (Figure 7A), encoding proteins IscS, IscU, IscA, Hsc66, Hsc20, and ferredoxin responsible for Fe-S assembly. The length of the *isc* operon was 5664 bp, the length of *iscR* was 537 bp encoding a transcriptional regulator (178 aa protein). Insertion sites of iscR-280, iscR-327, and iscR-513 were located at 280 bp, 327 bp and 513 bp of *iscR*, respectively, as well as insertion sites of iscR-280, iscR-327 located at the predicted function domain of Rrf2 (Figure 7B). The gap between *iscR* and *iscS* was 78 bp, and insertion site of mutant iscS + 30 was located at 48 bp downstream of *iscR* and 30 bp upstream of *iscS*. In Fe-S cluster assembly pathway, IscS is a cysteine desulfurase that procures the sulfur from cysteine for Fe-S cluster assembly [27]; IscR is an iron-sulphur (Fe-S) cluster containing transcription factor that represses transcription of the *isc* operon in *E. coli*, but *iscRSUA* operon was induced under oxidative stress [28],[29]. In other bacteria, IscR was shown to both behave an activator or a repressor.

The insertional mutants were more sensitive to high concentrations of Se(IV) than *C. testosteroni* S44 and also grew more slowly in 10 mM Se(IV) than wild type *C. testosteroni* S44 . Se(IV) reduction of iscR-513 and iscS + 30 was also delayed but not as much as iscR-280 and iscR-327 (Figure 7C). The growth of iscR-280 and iscR-327 was completely inhibited in 50 mM Se(IV), whereas *C. testosteroni* S44, iscR-513 and iscS + 30 showed slow growth and decreased Se(IV) reduction. Those results indicated that iscR-327 was the most sensitive mutant to higher concentrations of Se(IV), followed by iscR-280 with intermediate sensitivity in iscR-513 and iscS + 30, and the highest resistance in wild type *C. testosteroni* S44. Despite of different resistance between wild type and *iscR* mutants, the presence of IscR was not essential for Se(IV) reduction. For example, in 10 mM Se(IV), iscR-280 and iscR-327 grew slowly with little apparent Se(IV) reduction and showed faint red color after 12 and 16 h incubation; in contrast, the red color due to selenium nanoparticles became similar to the wild type after 24 h incubation, indicating IscR was necessary for the growth and resistance but was not necessary for Se(IV) reduction to occur.

**Figure 8:**
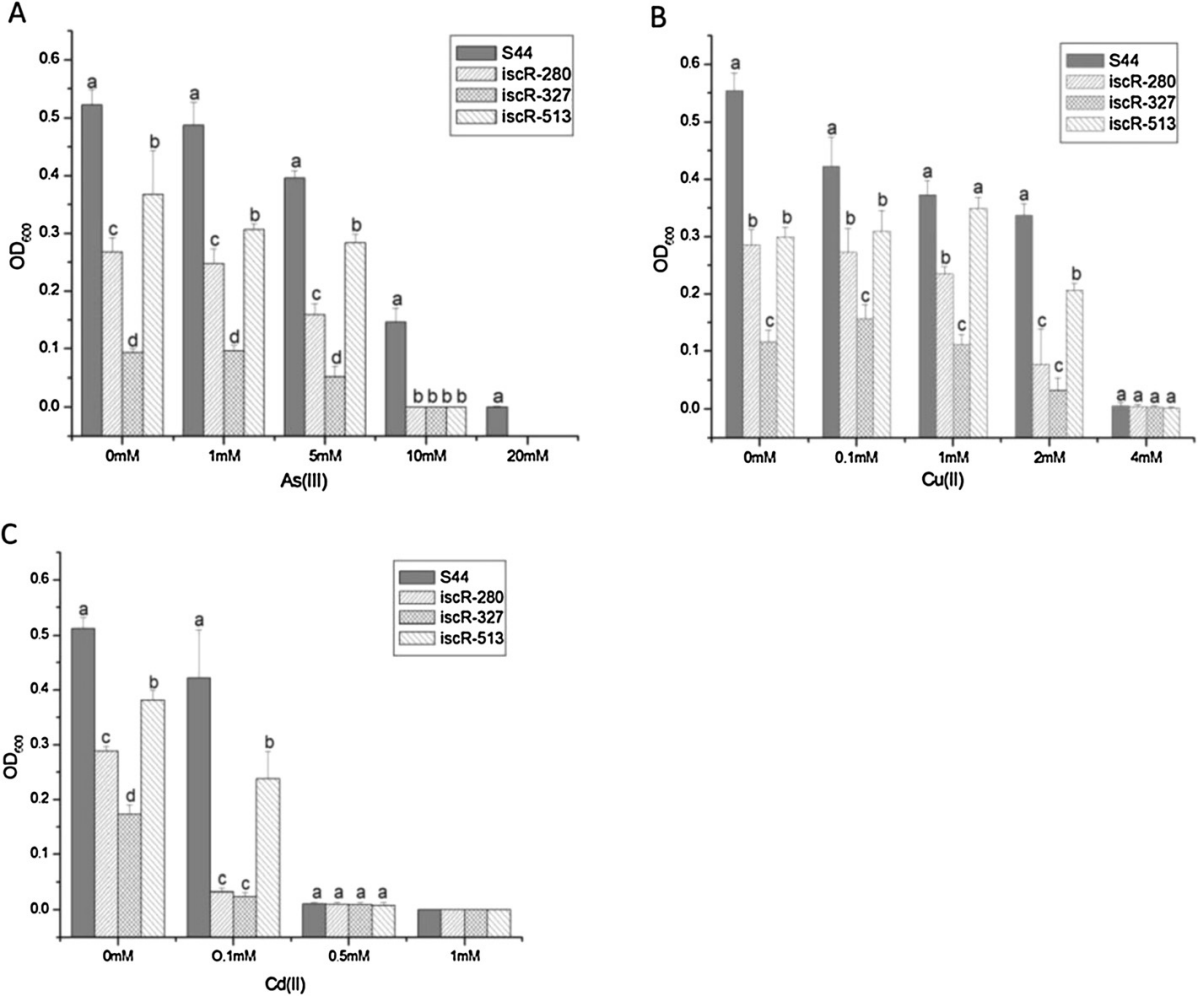
**Resistance of*C. testosteroni*S44 and*iscR*mutants to As(III), Cu(II) and Cd(II).** All strains were inoculated into 5 ml liquid LB medium supplemented with different concentrations of **(A)** As(III), **(B)** Cu(II) and **(C)** Cd(II), respectively. The OD value was determined after 24 h incubation. Different letters above bars at each metal concentration indicate significant differences between wild type S44, mutants iscR-280, iscR-327 and iscR-513 (*P* < 0.05).

In order to understand whether IscR influenced resistance to other heavy or transition metal(loid)s, we determined the growth of *iscR* mutants and the wild type. The wild type *C. testosteroni* S44 grew better than three *iscR* mutants iscR-280, iscR-327 and iscR-513 under heavy metal(loid)s such as As (III), Cu (II) and Cd (II) (Figure 8). Most sensitive to all metals and metalloids was insertional mutant iscR-327, followed by iscR-280 and iscR-513. In addition, the MICs of As (III), Cu (II) and Cd (II) in wild type *C. testosteroni* S44 were 20 mM, 4 mM and 0.5 mM, respectively. In contrast, the MICs of As (III), Cu (II) and Cd (II) in mutants iscR-280 and iscR-327 decreased to 10 mM, 2 mM and 0.1 mM, respectively. Those results indicated that IscR was involved in conferring resistance to a number of transition, heavy metals and metalloids in *C. testosteroni* S44.

## Discussion

*C. testosteroni* S44 reduced soluble Se(IV) into insoluble and thus non-toxic SeNPs outside of cells under aerobic condition as indicated by SEM/TEM-EDX and EDS Mapping analyses. It should thus be possible to synthesize SeNPs by imitating the biological process in industrial nanomaterial manufacturing [30]. Diseases caused by high content of Se in soils have been confirmed for the Chinese provinces Hubei and Shaanxi and Indian Punjab [1],[4]. In general, the variation of Se level in humans and animals are correlated to both Se excess and deficiency through the food chain [20]. Plants took up less water-soluble Se oxyanions from soil when bacteria reduced Se(IV) to organic Se and element selenium [31]. High levels of Se are commonly associated with concurrent contamination by other heavy and/or transition metals. Therefore, *C. testosteroni* S44 could be very useful for bioremediation of heavy metal(loid) polluted soils because it has adapted to a metal(loid)-contaminated environment. Considering the fact that only a partial reduction of Se(IV) to Se(0) could be achieved (Figure 2), it would be better in Se bioremediation if *C. testosteroni* S44 was applied to the contaminated site together with other more efficient Se(IV)-reducing bacteria.

In some bacterial strains, elemental SeNPs were observed both inside and outside of cells [12],[21],[32],[33] whereas in other bacteria nanoparticles were only observed outside of cells [20]. We did not detect Se(IV) by HPLC-HG-AFS in cellular fractions (data not shown) although elemental Se less than 0.1 μM meets the demand of bacteria for synthesis of selenocysteine [34]. We could not observe SeNPs produced inside of cells at log phase and stationary phase by TEM, EDX and EDS Elemental Mapping (Figures 3, 4 and Additional file 1: Figure S1) although SeNPs were easily observed by TEM in many bacterial cells [12],[21],[32]. In contrast, we only observed a large number of SeNPs appearing outside of cells (Figure 1). The cytoplasmic fraction showed the strongest Se(IV) reducing ability contrasting with a weak reducing ability in periplasmic and membrane fractions after addition of NADPH (Figure 6) or NADH (Data not shown). Accordingly, the process of Se(IV) reduction appears to be an NADPH- or NADH-dependent pathway and indicates two possible pathways. One possibility is that Se(IV) did not enter the cytoplasm of strain S44 or only trace levels of Se(IV) were present in the cytoplasm. The Se(IV)-reducing determinant might have initially been assembled in the cytoplasm and then transferred across cytoplasmic and outer membrane. The Se(IV)-reducing determinant would then be only active outside of cells *in vivo*[21]. Another possibility, and more likely at that, is that Se(IV) was reduced to Se(0) in the cytoplasm and then Se(0) was pumped out of the cells where small SeNPs aggregated into bigger particles.

In many cases, the big and smooth-surface nanoparticles occurred outside of cells [20],[21],[32]. Here, a large number of SeNPs ranging from 100–200 nm were observed by SEM (Figure 1) and further confirmed by EDX (Figure 3A). In our experiment it was obvious that small selenium particles aggregated into bigger particles as observed by TEM (Figure 3 and Additional file 1: Figure S1). This was different from previous TEM images of a homogeneous density of SeNPs [20],[21],[32]. In addition, this was not impacted by sample preparation because other strains produced big and homogeneous nanoparticles outside of cells using the same sample preparation and TEM observation technique (Data not shown). Previous studies confirmed small particles having low negative charges to have a propensity to come together and form aggregates [12]. In addition, proteins and/or other biomolecules such as polysaccharides and fatty acid may play a key role in controlling selenium nanoparticle size and the morphology of the resultant SeNPs [30]. The bulk of the Se(VI) and Se(IV) reduction to Se(0) was reported to occur on or outside the envelope [21]. This is very different from the reported mechanism where selenium was bound to the assembling protein SefA and then formed nanoparticles which were exported from cells [35].

In most reported cases, Se(VI) reduction occurred under anaerobic condition [36]-[38]. *C. testosteroni* S44 has a weak ability to reduce Se(VI) into red-colored selenium under aerobic condition (Figure 5B). The Se(VI) reductase complex was identified as a periplasmic Mo-containing enzyme in *T. selenatis*[38],[39] and *B. selenatarsenatis*[40]. The Se(VI)-reducing determinant of *C. testosteroni* S44 also is most likely a Mo-enzyme because tungstate inhibited Se(VI) reduction (Figure 5B). In contrast, the Se(IV)-reducing determinant did not appear to contain Mo because tungstate did not inhibit Se(IV) reduction. Accordingly, Se(VI) reduction is a distinct activity different from Se(IV) reduction.

Iron-sulfur (Fe-S) clusters are cofactors for many proteins across all three domains of life. Fe-S proteins function in a number of cellular processes, including electron transfer, gene regulation, photosynthesis and nitrogen fixation, anti-oxidative and iron stress among others [28],[29],[41]. The genomic organization of *iscRSUA-hscBA-fdx*, the operon encoding the housekeeping Fe-S biogenesis system (Isc), is conserved in many β- and γ-proteobacteria [27]. IscR (Isc regulator) regulates expression of the Isc pathway by modulating intracellular iron homeostasis via a negative feedback mechanism based on the cellular Fe-S demand in *P. aeruginosa* and *E. coli*[42],[43] and can also increase the expression of another operon, *sufABCDSE,* involved in synthesis of Fe-S clusters in *E. coli*[28],[29],[41]. IscR is part of the large Rrf2 family of winged helix-turn-helix (wHTH) transcription factors [44]. We could not find a *suf* operon on the genome of *C. testosteroni* S44, this is similar to genome of *Pseudomonas* spp. that is also lacking a *suf* operon [43]. As a result, only *iscRSUA-hscBA-fdx* encoding proteins are used for Fe-S cluster synthesis in *C. testosteroni* S44. In addition, IscR is a global regulator that regulates functions not only involved in Fe-S biogenesis but also directly or indirectly controlling the expression of ~40 genes in *E. coli*[28],[41]. Recently, it was shown that the highly conserved three cysteine residues (Cys92, Cys98, and Cys104) and His107 of IscR were essential for [2Fe-2S] cluster ligation [45]. [2Fe-2S]-IscR binds both type 1 and type 2 motifs from *hya* promoter, thereby exhibiting metal-dependent regulation of DNA binding specific for IscR [46]. The corresponding cluster ligands are Cys92, Cys98, Cys105 and His108 in IscR from *C. testosteroni* S44. The insertion sites of Tn5 mutants, iscR-280 and iscR-327, were close to bases encoding those four ligands. Moreover, the insertion site of iscR-327 was located next to the bases encoding His108 located at residues forming a helix involved in dimerization (residues 103–123 in *E. coli*) of IscR [46], therefore disturbing the formation of IscR dimers. In contrast, the insertion site of iscR-513 is located at the tail end of *iscR* (537 bp full length) and the insertion site in iscS + 30 is located at the gap between *iscR* and *iscS* (Figure 7). As a result, the formation and function of IscR were more strongly disturbed in iscR-280 and especially in iscR-327, resulting in slower growth and less resistance than iscR-513 to heavy metal(loid)s (Figures 7 and 8). The insertional mutants iscR-513 and iscS + 30 would still produce a functional IscR regulator (albeit truncated at the C-terminus in iscR-513) but expression of subsequent genes of the operon would be significantly lower due to polar effects of an insertion by transposon Tn5. Those results are consistent with the result of a *∆iscR* mutant that was 40- to 50-fold less resistant to organic hydroperoxides (tBOOH and CuOOH) in *P. aeruginosa*[43]. Therefore, IscR aids cellular growth and resistance to heavy metals not only by regulating expression of the *iscSUA-hscBA-fdx* operon, but probably also by directly or indirectly regulating expression of other genes [28] in *C. testosteroni* S44.

*C. testosteroni* S44 was isolated from an antimony mine and contained resistance determinants to various metal(loid)s [26]. Due to a large number of genes encoding putative metal(loid) resistance proteins [26], *C. testosteroni* S44 is thought to be able to quickly pump heavy or transition metals and metalloids out of the cell or transform them into a less toxic species thereby becoming very resistant. This interpretation is consistent with the high MIC for Se(IV) and the postulated quick Se(0) secretion from the cytoplasm across the cell envelope to the outside of cells. Although *C. testosteroni* S44 was resistant to high level of heavy metals, it did not reduce Se(IV) efficiently. It is therefore possible *C. testosteroni* S44 evolved a balanced state between resistance of Se oxyanions and reduction (detoxification).

## Conclusion

A strict aerobic bacterium, *C. testosteroni* S44, reduced Se(VI) and Se(IV) to red SeNPs with sizes ranging from 100 to 200 nm. The cytoplasmic fraction strongly reduced Se(IV) to red-colored selenium in the presence of NADPH but no SeNPs were observed in cells. Possibly, Se(IV) was reduced in the cytoplasm and then transported out of the cell where the SeNPs were formed.

## Methods

### Growth, Se(IV) resistance and reduction tests of *C. testosteroni* S44

*C. testosteroni* S44 was inoculated in a 96 well plate with LB liquid medium with different concentrations of Se(IV) added to determine the minimal inhibitory concentration (MIC). Cells were incubated at 28°C with shaking at 180 rpm under either aerobic or anaerobic conditions.

For determination of a growth curve, *C. testosteroni* S44 was inoculated into 100 ml liquid LB medium supplemented with different concentrations of sodium selenite ranging from 0.2 mM to 25.0 mM and incubated at 28°C with shaking at 180 rpm. Cultures were taken every 4 h to measure growth based on the cellular protein content by an EnVision® Multimode Plate Reader (Perkin Elmer) as described in Bradford [47] and Binks et al. [48]. Se(IV) concentrations were measured by HPLC-HG-AFS (Beijing Titan Instruments Co., Ltd., China) as described in Li et al. [49].

### Scanning Electron Microscopy (SEM)

*C. testosteroni* S44 was grown in LB supplemented with 1.0 to 20 mM sodium selenite at 28°C. After 24 h of incubation, cells were centrifuged (6,000 rpm, 10 min, 4°C) and SEM observation was performed on the processed samples. Sample processing involves washing, fixing and drying of cells at 4°C. Harvested cells were washed thrice with phosphate buffer saline (PBS, pH7.2). Fixation was done with 2.5% glutaraldehyde (24 h, 4°C). Fixed cells were dehydrated through a series of alcohol dehydration steps (30%, 50%, 70%, 85%, 95% and 100%) and finally freeze dried and sputter coated. The samples were then viewed using SEM.

### Transmission electron microscopy (TEM) and Energy Dispersive Spectroscopy (EDS/EDX) Elemental Mapping

*C. testosteroni* S44 was cultured in LB broth with 1 mM Se(IV) at 26°C with shaking at 180 rpm, harvested at both log phase and stationary phase. Samples that were grown without Se(IV) were used as controls. Cultured samples were fixed using 2% v/v glutaraldehyde in 0.05 M sodium phosphate buffer (pH 7.2) for 24 h and were then rinsed three times in 0.15 M sodium cacodylate buffer (pH 7.2) for 2 h. The specimens were dehydrated in graded series of ethanol (70%, 96% and 100%) transferred to propylene oxide and embedded in Epon according to standard procedures. Sections, approximately 80 nm thick, were cut with a Reichert-Jung Ultracut E microtome and collected on copper grids with Formvar supporting membranes. The sections were stained or unstained with uranyl acetate and lead citrate and then TEM-STEM-EDX (TITAN 120 kV) and EDS Mapping (QUANTA 200 F) were performed, respectively.

### Tungstate test on Se(IV) and Se(VI) reduction

*C. testosteroni* S44 cells were incubated in CDM (chemically defined medium) [50], LB and TSB plates supplemented with 0.2 mM sodium selenite, 5.0 mM sodium selenate, respectively, and with or without 10 mM tungstate (Na_2_O_4_W.2H_2_O) at 26°C under aerobic condition for two days. The inhibiting effect of tungstate was shown by appearance or absence of the specific red color of SeNPs in comparison with control in absence of tungstate.

### Cellular fractionations and determination of Se(IV)-reducing activity

Log-phase (12 hr) and stationary phase (20 hr) cells of *C. testosteroni* S44 were obtained by growth at 26°C, shaking at 180 rpm in 20 ml LB broth. The modified method was based on protocol of method No. 5 for subcellular fractionation [51]. All further parts of the procedure were carried out at 0 to 4°C unless differently noted.

The cells in 20 ml LB cultures were harvested by centrifugation for 20 min at 4,500 × g, and then the supernatant was removed. After being harvested, the cells were suspended in 2.0 ml 1 × PBS buffer (pH 7.0), centrifuged three times for 10 min at 4,500 × g. The cells were then suspended in 1.0 ml 1 × PBS buffer (pH 7.0) containing 5% glycerol (v/v, final concentration). The suspension was treated with 1.0 mg ml^−1^ (final content) lysozyme for 5 min at room temperature and afterwards centrifuged for 20 min at 20,000 × g. The supernatant was periplasmic protein. In order to separate the membranes from the cytoplasm, the pellet was suspended in 1.0 ml 1 × PBS buffer containing 5% glycerol (v/v) and 125 units per ml (final concentration) DNase I. The suspension was treated with ultrasound for 20 min (20 amplitude microns, 5 s /5 s, Sanyo Soniprep). The broken-cell suspension was centrifuged for 6 min at 6000 × g to remove unbroken cells. The supernatant was centrifuged for 60 min at 20,000 × g. The supernatant contained the cytoplasmic fraction and the pellet contained the crude membranes (outer membrane and cytoplasmic membrane).

Se(IV)-reducing activity was estimated by the accumulation of red SeNPs after either the periplasmic fraction, membranes or the cytoplasmic fraction were incubated at 26°C in 1 × PBS buffer containing 5% glycerol, 0.2 mM Se(IV) and 0.2 mM NADPH, respectively.

### Transposon mutagenesis and screening of mutants defective for Se(IV) resistance and reduction

*E. coli* strain S17-1(pRL27-Cm) was used as the donor strain for transposon Tn5, and *C. testosteroni* S44 was used as the recipient. Plasmid pRL27-Cm was transferred into the recipients *C. testosteroni* S44 by conjugation from *E. coli* strain S17-1 carrying Tn5 according to the method of Larsen [52]. Selection was carried out on LB agar plates containing 50 μg ml^-1^ chloramphenicol (Cm) and 50 μg ml^-1^ rifampin (Rif). To obtain the sensitive strains for Se(IV) the colonies of mutants from the mating plates were inoculated onto LB agar plates with 50 μg ml^-1^ Cm, 50 μg ml^-1^ Rif, 50 mM Se(IV) using sterile toothpicks, incubated at 28°C for 1–2 days to allow the colonies to reduce Se(IV) and develop the red colored SeNPs indicative of elemental selenium. The wild type *C. testosteroni* S44 was used as control. Se(IV) sensitive strains were screened for slow growth, death or less red in the medium containing 1 mM and 50 mM Se(IV). Then sensitive mutants were restreaked on LB medium with 1 mM and 50 mM Se(IV), respectively to further confirm the phenotype of Se(IV) reduction and resistance. Bacterial strains and plasmids used in this study were shown as Table 1.

**Table 1:** Bacterial strains and plasmids used in this study

**Strain or plasmid**	**Relevant properties or derivation**	**Source or reference**
*C. testosteroni* S44	Wild type, Rif^r^, Cm^s^, Tet^s^	[26]
iscR-280, iscR-327, iscR-513	*iscR* Tn5 insertional mutants	This study
	Rif^r^, Cm^r^, Tet^s^	
iscS + 30	Tn5 insertional mutant downstream of *iscR*, Rif^r^, Cm^r^, Tet^s^	This study
*E. coli* S17-1(λ*pir*)	*Tpr Smr recA thi pro hsdR*^*−*^*hsdM*^*+*^. RP4:2Tc:Mu:Km T7, λ*pir*	Lab collection
Plasmids		
pRL27-Cm	Transposon vector , *ori*R6K , Cm^r^	[52]
pCPP30	Broad host range, *tetA*	Timothy R. McDermott, Montana State University

### Inverse PCR, DNA sequencing and analysis

The chromosomal DNA adjacent to the sites of Tn5 insertion was determined in individual mutants by inverse PCR using primers pRLSR (5′-AACAAGCCAGGGATGTAACG-3′) and pRLSF (5′- CAGCAACACCTTCTTCACGA -3′) which were designed outwardly within the transposon. The DNA of each mutant was extracted using phenol-chloroform and then digested with *Bgl*II (Fermentas) which does not cut within the transposon. Subsequently, the digested DNA was self-ligated in a 30 μl reaction with 6U of T4 DNA ligase (Promega) and transferred into *E. coli* strain S17-1(λ*pir*), where circularized DNA containing flanking fragments of the site of Tn5 insertion and transposon replicate as a plasmids. Transposon junction plasmids were isolated from selected transformants and subjected to inverse PCR using primers pRLSR and pRLSF which anneal to the *ori*R6K and Cm^r^ ends of the transposon, respectively. The PCR products were purified using the Gel Extraction kit (Watson Biotechnologies, China) and sequenced. Sequences were analysed using the BlastX algorithm [53] compared to the protein sequence database (GenBank).

### Growth measurement in presence of different concentrations of metal(loid)s

The wild type strain *C. testosteroni* S44, *iscR* mutants *C. testosteroni* iscR-280, iscR-327 and iscR-513, and a mutant of *iscR* downstream, iscS + 30, were inoculated into 5 ml liquid LB medium supplemented with differing concentrations of Se(IV) encompassing 10.0, 25.0, 50.0 and 100.0 mM, respectively at 28°C with shaking at 180 rpm. Likewise, the wild type strain and four mutants were inoculated into 5 ml liquid LB medium supplemented with As(III), Cu(II) and Cd(II), respectively. The concentrations of As(III) were 0, 1.0, 5.0, 10.0, 20.0 mM, for Cd(II) they were 0, 0.1, 0.5, 1.0 mM, and for Cu(II) they were 0, 0.1, 1.0, 2.0, 4.0 mM, respectively. Cells were incubated at 26°C with shaking at 180 rpm. The OD_600_ value was determined after 24 h incubation.

## Competing interests

The authors declare that they have no competing interests.

## Authors’ contributions

SZ, CR and GW designed the experiments. SZ conducted the experiments including EDX, EDS Mapping, TEM, subcellular fraction, resistance of heavy metals, and tungstate test, analyzed the results and wrote the manuscript. JS performed transposon mutagenesis and Se(IV) resistance. LW, RY, DW and RW conducted SEM, growth and Se(IV) reduction curves. YD assisted to EDS Mapping. CR and GW reviewed and revised the manuscript. All authors read and approved the final manuscript.

## Additional file

## Supplementary Material

Additional file 1: Figure S1. TEM graphs of *C. testosteroni* S44 amended with 1.0 mM Se(IV) at different times of incubation. **B** and **D**, strain S44 amended with Se(IV) at log phase and stationary phase, respectively. **A** and **C** are control (no Se(IV) ) at log phase and stationary phase, respectively. Arrows indicated extracellular selenium particles. Click here for file
