# Supplementary figures and images for: Selenite reduction by the obligate aerobic bacterium Comamonas testosteroni S44 isolated from a metal-contaminated soil
Source: BMC Microbiol. 2014 Aug 7;14:204. doi: 10.1186/s12866-014-0204-8 (PMC4236595; doi:10.1186/s12866-014-0204-8)

**Additional file: Figure S1**

**
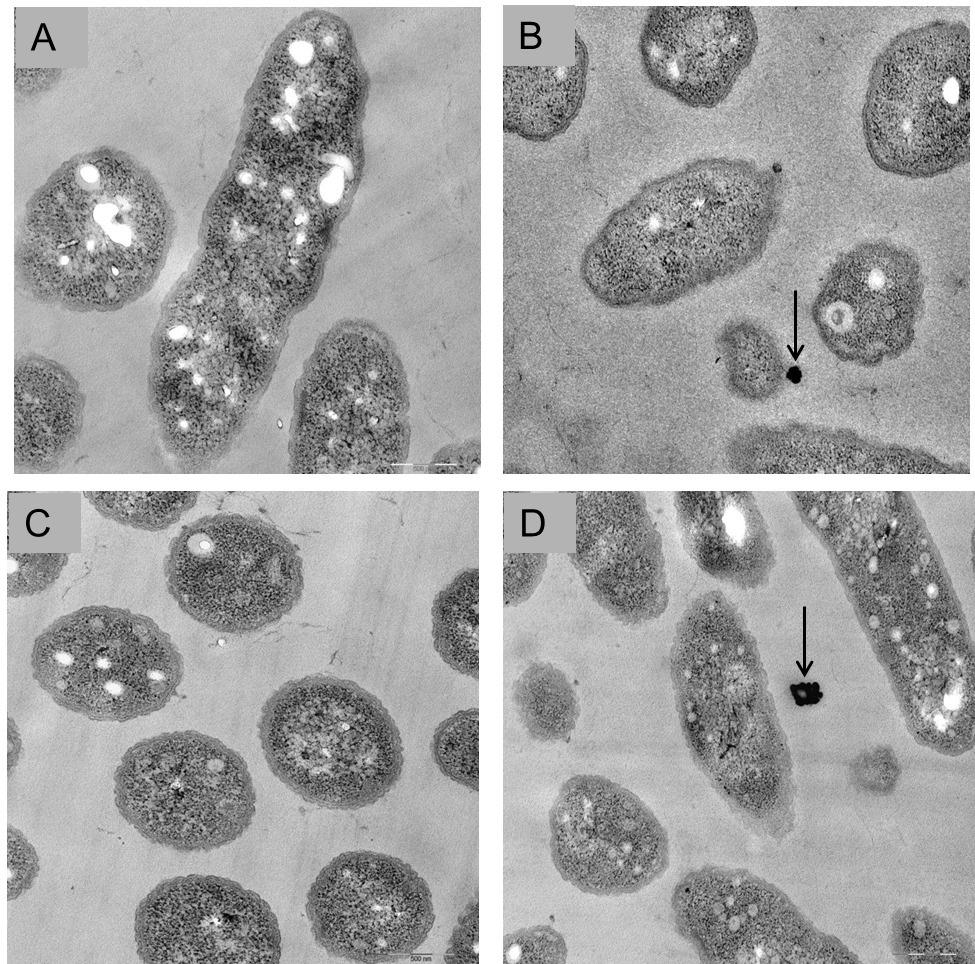
**

Supplement: Additional file 1: Figure S1. — TEM graphs of C. testosteroni S44 amended with 1.0 mM Se(IV) at different times of incubation. B and D, strain S44 amended with Se(IV) at log phase and stationary phase, respectively. A and C are control (no Se(IV) ) at log phase and stationary phase, respectively. Arrows indicated extracellular selenium particles. [file s12866-014-0204-8-S1.docx]
